# On the necessity of different statistical treatment for Illumina BeadChip and Affymetrix GeneChip data and its significance for biological interpretation

**DOI:** 10.1186/1745-6150-3-23

**Published:** 2008-06-03

**Authors:** Wing-Cheong Wong, Marie Loh, Frank Eisenhaber

**Affiliations:** 1 Bioinformatics Institute (BII), Agency for Science, Technology and Research (A*STAR), 30 Biopolis Street #07-01, Matrix Building, 138671, Singapore

## Abstract

**Background:**

The original spotted array technology with competitive hybridization of two experimental samples and measuring relative expression levels is increasingly displaced by more accurate platforms that allow determining absolute expression values for a single sample (for example, Affymetrix GeneChip and Illumina BeadChip). Unfortunately, cross-platform comparisons show a disappointingly low concordance between lists of regulated genes between the latter two platforms.

**Results:**

Whereas expression values determined with a single Affymetrix GeneChip represent single measurements, the expression results obtained with Illumina BeadChip are essentially statistical means from several dozens of identical probes. In the case of multiple technical replicates, the data require, therefore, different stistical treatment depending on the platform. The key is the computation of the squared standard deviation within replicates in the case of the Illumina data as weighted mean of the square of the standard deviations of the individual experiments. With an Illumina spike experiment, we demonstrate dramatically improved significance of spiked genes over all relevant concentration ranges. The re-evaluation of two published Illumina datasets (membrane type-1 matrix metalloproteinase expression in mammary epithelial cells by Golubkov et al. Cancer Research (2006) 66, 10460; spermatogenesis in normal and teratozoospermic men, Platts et al. Human Molecular Genetics (2007) 16, 763) significantly identified more biologically relevant genes as transcriptionally regulated targets and, thus, additional biological pathways involved.

**Conclusion:**

The results in this work show that it is important to process Illumina BeadChip data in a modified statistical procedure and to compute the standard deviation in experiments with technical replicates from the standard errors of individual BeadChips. This change leads also to an improved concordance with Affymetrix GeneChip results as the spermatogenesis dataset re-evaluation demonstrates.

**Reviewers:**

This article was reviewed by I. King Jordan, Mark J. Dunning and Shamil Sunyaev.

## Background

Microarrays that rely on hybridization with DNA probes pioneered large-scale expression studies. After the introduction of spotted array technology in the mid 90s, microarrays have steadily gained popularity for exploratory gene expression analysis. A spotted array experiment requires both the treated and control samples to be labeled with different dyes and to be competitively hybridized on the same array. The expression level is expressed as a ratio between the intensities between the two labels. Spotted arrays are plagued by accuracy and sensitivity problems that are only partly remedied by the measuring only relative expression. Dye bias and repeatability remain unsatisfactory.

In recent years, Affymetrix GeneChip and Illumina BeadChip have emerged as two of the most popular microarray platforms. From the experimental design viewpoint, the GeneChip and BeadChip offer flexibility in terms of their ability to measure absolute expression values for each experimental sample independently. The growing amount of publicly available microarray data has prompted researchers to explore ways to compare results between experiments across the different platforms. This task signifies the first step in producing consistent and trusted results to support meaningful biological discovery. Yet, it is more difficult than it appears superficially. Even a simple variant of the problem like comparing results from the same sample across different platforms is not trivial. The first step requires the statistically significant changes in gene expression to be determined between treatment conditions for each platform. The platform-specific gene lists generated by applying the same significance threshold are finally compared. In general, the concordance between these gene lists is disappointingly low. Nevertheless, recent works have shown that concordance improvements can be made by filtering for gene nucleotide sequence identity [1-4], by suppressing lower intensity genes [5] or by aligning gene lists with continuous measures of differential gene expression [6].

During our evaluation of cross-platform comparison between Affymetrix and Illumina, we stumbled upon another, quite surprising reason for the low concordance. Given the specific design of Illumina arrays [7-10], it appears that the data derived from them requires specific statistical treatment different from that of more classical microarrays. Notably, the Affymetrix GeneChip and Illumina BeadChip have one stark difference in their designs. In a nutshell, many instances of a unique probe design are synthesized onto a group of adjacent discrete features or cells on the GeneChip. Consequently, each group of cells will target a particular gene. In the case of Illumina BeadChip, a unit of bead coated with hundred of thousands of probes is analogous to a group of cells on GeneChip. Furthermore, multiple beads of a probe design are immobilized onto randomized positions on the BeadChip. Therefore, given a probe design, a gene is only measured once on the GeneChip, whereas it is measured typically about 30 times on the Beadchip. But instead of delivering the individual bead intensities (possible with appropriate scanner modifications), the mean and standard error (i.e., the standard deviation divided by the square root of the number of beads) of the bead intensities, known as the summary data, are usually reported.

Thus, Affymetrix GeneChips provide individual measurement results but the Illumina BeadChips generate means and standard errors for subsets of bead intensity measurements. Therefore, the summary data of a BeadChip experiment requires a different statistical interpretation compared with the individual measurements in the case of GeneChip data, especially in cases of multiple technical replicates. If the average of the bead intensities delivered by a single Illumina BeadChip is fed into standard expression profile analysis software (for example, GeneSpring), the standard deviation over technical replicates is calculated from the deviations of the subset means from the overall mean. But more correctly, the overall standard error is to be computed by taking into account also the standard deviations obtained from the individual BeadChips.

In this paper, we will first present a derivation for the correct summary statistic applicable to Illumina BeadChips data. Furthermore, this summary statistic will be applied to one control experiment with artificial spikes and, also, it will be used for the re-evaluation of two published biological experiments. In all cases, the modified treatment is contrasted against the standard one. In the control experiment [11], we will demonstrate a dramatic improvement in recognizing the spike sequence selection if the corrected summary statistic is applied. In the example of the MT1-MMP mammary epithelium dataset [7], cell cycle pathway involvement can be shown with statistical confidence only after applying the correct summary statistic. Interestingly, cell cycle gene involvement was suggested by the authors, although their analysis of the data did not provide strong arguments for it. Then with the spermatogenesis cross-platform data [8], we demonstrate that considerably improved concordance between the Affymetrix and Illumina platforms can be achieved with the correct summary statistic. Our analysis also provides new evidence for the transcriptional regulation of the N-glycan biosynthesis, the tight and the adherens junction pathways in this context, a finding that is supported also by independent experimental evidence.

## Results & Discussion

### Statistics of Illumina BeadChip & Affymetrix GeneChip datasets

The bead intensity of a given gene in a BeadChip is described with the random variable *X*. The expression profile experiment is supposed to consist of *K *technical replicates (independent measurement of arrays on the same biological sample). Each bead intensity *x*_*k*,*n *_is an instance of the random variable *X *(where *k *= 1...*K *replicates, *n *= 1...*N*_*k *_beads, *N*_*k *_is the number of beads in the *k*-th technical replicate). We assume that the first *M*_*k *_beads are retained after outlier removal (see below). The summary data includes the mean *μ*_*k*_, the standard error $\sigma_{k}/\sqrt{M_{k}}$ (where *σ*_*k *_is the standard deviation) and the number of beads *M*_*k *_(the typical value of *M*_*k *_is about 30).

The observed BeadChip intensities of gene in the *k*-th array are denoted as $\bar{X_{k}}={\left[\begin{array}{cccc} x_{k,1} & x_{k,2} & \cdots & x_{k,N_{k}} \end{array}\right]}^{T}$ (Table 1). However, a typical BeadChip experiment does not report these individual bead intensities. Instead, the Illumina BeadStudio software first performs an outlier removal on the bead intensities. Instances with intensities above three median absolute deviations from the median are removed. Upon the outlier removal, the mean and standard error of the bead intensities as well as the number of beads used in summarization for each gene are reported (the AVG_signal, BEAD_STDERR, Avg_NBEADS columns in the Illumina Beadstudio output file).

**Table 1:** Intensity output of Illumina & Affymetrix across *K *technical replicates

Platform	Replicate 1	Replicate 2	⋯	Replicate K
Illumina BeadChip (Raw data)	$\bar{X_{1}}=\left[\begin{array}{c} x_{1,1}\\ x_{1,2}\\ \vdots \\ x_{1,N_{1}} \end{array}\right]$	$\bar{X_{2}}=\left[\begin{array}{c} x_{2,1}\\ x_{2,2}\\ \vdots \\ x_{2,N_{2}} \end{array}\right]$	⋯	$\bar{X_{K}}=\left[\begin{array}{c} x_{K,1}\\ x_{K,2}\\ \vdots \\ x_{K,N_{K}} \end{array}\right]$
Illumina BeadChip (Summary data)	$\mu_{1},\frac{\sigma_{1}}{\sqrt{M_{1}}},M_{1}$	$\mu_{2},\frac{\sigma_{2}}{\sqrt{M_{2}}},M_{2}$	⋯	$\mu_{K},\frac{\sigma_{K}}{\sqrt{M_{K}}},M_{K}$
Affymetrix GeneChip (Raw data)	*x*_1,1_	*x*_2,1_	⋯	*X*_*K*,1_

Using the means and standard errors of all the technical replicates, the mean *μ*_*total *_and standard deviation *σ*_*total *_of bead intensities of a gene across the *K *technical replicates are given as

(1) $$\mu_{total}=\frac{1}{K}\sum_{k=1}^{K}\mu_{k}$$

(2) $$\sigma_{total}=\sqrt{\sigma_{\mu }^{2}+\sigma_{wtrep}^{2}}$$

(3) $$\text{where }\sigma_{\mu }=\sqrt{\frac{1}{K}\sum_{k=1}^{K}\mu_{k}^{2}-{\left[\frac{1}{K}\sum_{k=1}^{K}\mu_{k}\right]}^{2}}$$

(4) $$\sigma_{wtrep}=\sqrt{\frac{M_{1}\sigma_{1}^{2}+M_{2}\sigma_{2}^{2}+...+M_{K}\sigma_{K}^{2}}{M_{1}+M_{2}+...+M_{K}}}$$

A proof for equation (2) is supplied in the Appendix 1. The standard deviation *σ*_*total *_is composed of two components each carrying a different meaning. Given that each of the *K *technical replicates represents the same, identical and independent distribution, one expects the *K *mean estimates *μ*_*k *_to be relatively similar and, hence, *σ*_*μ *_would be small relative to *σ*_*total *_and, ideally, close to zero. Since averaging for each replicate is carried out over about 30 individual measurements, it can be assumed that each individual *μ*_*k *_is likely a good estimate of the population mean *μ *if there were no batch variations. Therefore, a large *σ*_*μ *_can be interpreted as batch variation or noise among the replicates, a considerable part of which, apparently, has systematic origin such as variations in the total amount of hybridization-ready nucleic acids, etc. Ideally, *K *(typically 2–4) should be much larger in order to obtain a good estimate of *σ*_*μ*_. However, this is impractical due to the high cost of performing large number of microarrays. Therefore, we suggest to assume *σ*_*μ *_≈ 0 for the case of no batch variation in equation (5) and to use *σ*_*wtrep *_as a good lower estimate of the summary statistic in testing instead of *σ*_*μ *_(see Appendix 2 for proof).

(5) $$\sigma_{total}=\sqrt{\sigma_{wtrep}^{2}}$$

This proposed summary statistic is supported by observations communicated in two recent publications, which have leveraged on the variation in bead intensities. Dunning et al. [12] showed that differentially expressed gene detection experienced an increase in statistical power by using the inverse of $\sigma_{k}^{2}$ as weights in their linear model. On the other hand, Lin et al. [13] proposed a variance stabilization transformation that incorporated bead intensities variation and showed an improvement in differentially expressed gene detection. Beyond this point, we shall refer to *σ*_*total *_with respect to equation (5) instead of (2).

In the case of a Affymetrix GeneChip experiment, the measurement for a gene is taken only once in each replicate (see 4^th ^row of Table 1). Consequently, the mean and standard deviation of a gene across *K *technical replicates are given as

(6) $$\nu_{total}=\frac{1}{K}\sum_{k=1}^{K}x_{k,1}$$

(7) $$\omega_{total}=\sqrt{\frac{1}{K}\sum_{k=1}^{K}{\left(x_{k,1}-v_{total}\right)}^{2}}$$

The summary statistic [*μ*_*total*_, *σ*_*total*_] and [*ν*_*total*_, *ω*_*total*_] are the parallels between the Illumina and Affymetrix platforms. However, *σ*_*total *_has an advantage over *ω*_*total*_. Due to multiple copies of the same probe within a single Illumina array, the standard deviation can be computed for each array individually. As a result, *σ*_*total *_offers more protection against any systematic error than *ω*_*total *_(see Appendix 2 for proof). The lack of systematic error as a confounding factor in *σ*_*total *_increases the chance of detecting true biological differences from the statistical tests.

In any case, the more important concern related to the analysis of Illumina data is the mistake of treating the mean estimates of bead intensities as instances of the bead intensities. Standard gene expression profile analysis software (as applied in several published studies [7-10]) assumes that the imported data are bead intensities rather than mean estimates of bead intensities. Such a software plainly computes the mean and standard deviation for the incoming data and the corresponding summary statistic for the control and the treatment group would be [*μ*_*total*_, *σ*_*μ*_]_*control *_and [*μ*_*total*_, *σ*_*μ *_]_*treatment *_respectively. The summary statistic *σ*_*μ *_is incorrect since it measures only the batch variation and not at all variation in bead intensities. The correct summary statistic should be [*μ*_*total*_, *σ*_*wtrep*_]_*control *_and [*μ*_*total*_, *σ*_*wtrep*_]_*treatment*_. The emphasis of using *σ*_*wtrep *_instead of *σ*_*μ *_as the summary statistic is not statistical hair splitting but this issue affects the biological interpretation as we can see from the following three examples.

### Illumina spike data: improvement in p value ranking

Chudin et al. [14] provided Illumina BeadChip data for 34 spikes (varying concentrations from 0.01 to 1000 pM) against a background of human cRNA. To examine the effects of using *σ*_*μ *_instead of *σ*_*wtrep *_as summary statistic, each pair of spike experiments at adjacent concentrations were compared (Table 2). In each array, there are 34 spike and 48730 non-spike transcript probes. This is equivalent to 34 true alternative hypotheses and 48730 true null hypotheses. Ideally, the P-values of the 34 alternate hypotheses will appear on one extremity of the Schweder-Spojotvoll plot [15]. Upon computing and sorting the P-values of the pair-wise t-tests (after array normalization as in Materials and methods), each of the 34 smallest P-values was examined to see if it belongs to a true positive (TP) or false positive (FP) gene.

**Table 2:** Number of TP and FP genes based on P-value ranking.

	*σ*_*μ*_		*σ*_*wtrep*_		
		
**Concentration (in pM)**	**TP**	**FP**	**TP**	**FP**	**No. of common TP**
0.01 vs 0	0	34	0	34	0
0.03 vs 0.01	0	34	0	34	0
0.1 vs 0.03	7	27	9	25	7
0.3 vs 0.1	14	20	22	12	14
1 vs 0.3	30	4	33	1	30
3 vs 1	30	4	34	0	30
10 vs 3	33	1	34	0	33
30 vs 10	34	0	34	0	34
100 vs 30	33	1	34	0	33
300 vs 100	4	30	26	8	4
1000 vs 300	9	25	16	18	8

The first column indicates the 11 test cases of pair-wise treatment conditions. The second and third columns indicate the number of TP and FP genes respectively for using *σ*_*μ *_as the summary statistic. Similarly, column four and five indicate the results for using *σ*_*wtrep*_. The last column indicates the result for the number of common TP genes found by both *σ*_*μ *_and *σ*_*wtrep*_.

The number of identified TP genes by the statistic *σ*_*wtrep *_is generally higher than that by *σ*_*μ *_(Table 2). In particular, an improvement from 7 (0.1 and 0.03 pM comparison) or 14 (0.3 versus 0.1 pM) to 9 and 22 recovered spikes in the low concentration range of 0.03–0.3 pM is encouraging. Note that this region spans the endogenous gene expression level and, hence, it is critical to obtain good differentially expressed gene identification here. An improvement was also achieved in the high concentration region. But in practice, gene expression will not reach such level to leverage on it. Note that the detection limit was 0.25 pM while the saturation point was about 300 pM [11].

Most importantly, the TP genes found by *σ*_*μ *_is a subset of those found by *σ*_*wtrep*_. This means that more TP genes found by *σ*_*wtrep *_had moved into the first 34 ranks to displace only other FP genes. For that to happen, the P-values must have been re-ranked by the statistic so that the TP genes are more statistically significant than the FP genes.

**Figure 1:**
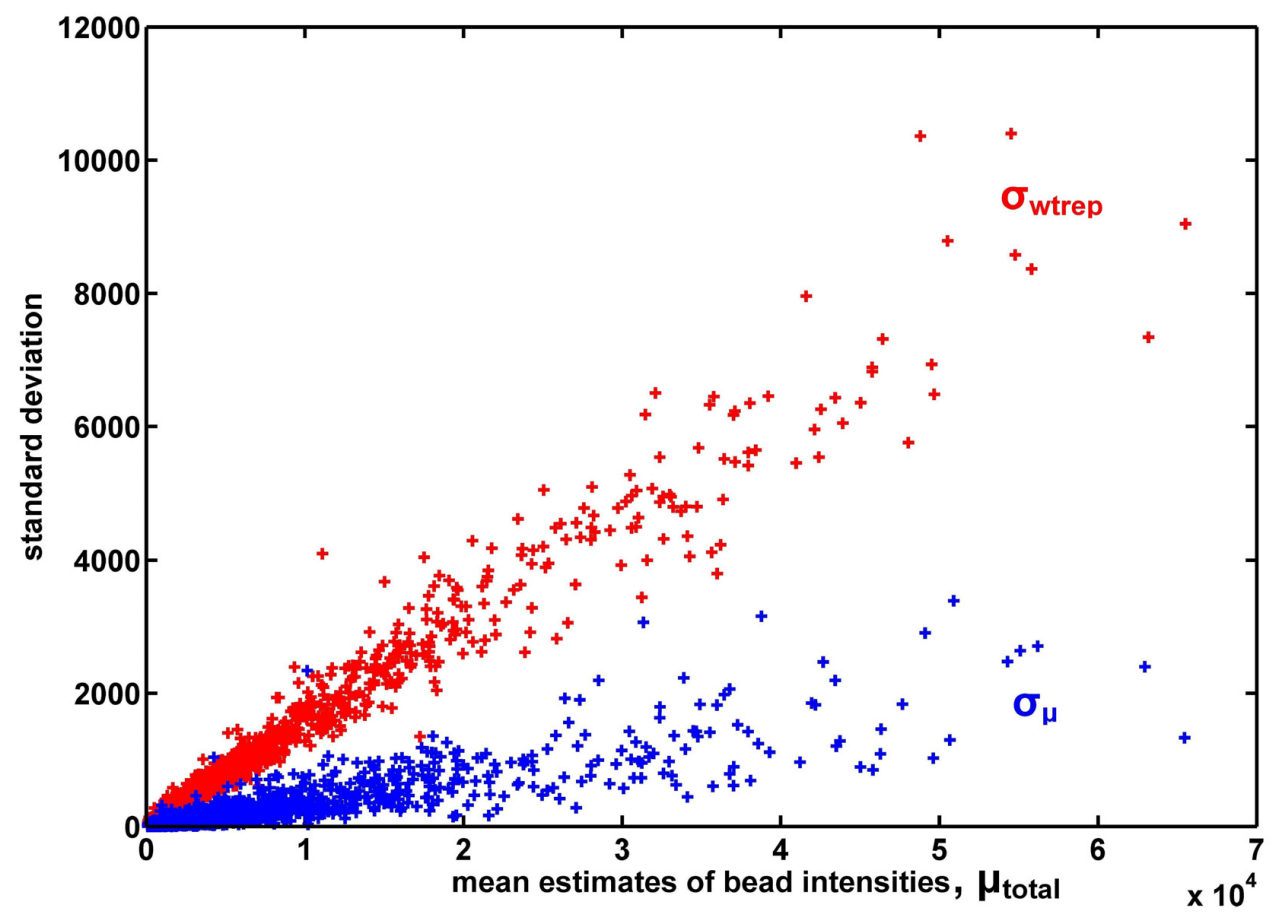
**Relationship between *σ*_*μ*_, *σ*_*wtrep *_and *μ*_*total*_**. We show the plot of mean estimates of bead intensities *μ*_*total *_against standard deviation in mean estimates *σ*_*μ *_and bead intensities *σ*_*wtrep *_of 0 pM spike concentration data.

This means that *σ*_*μ *_is not a good estimate for the standard deviation. Figure 1 shows a plot of mean bead intensities *μ*_*total *_against the standard deviation of mean estimates *σ*_*μ *_and variation in bead intensities *σ*_*wtrep *_of the human background cRNA (i.e. 0 pM spike data). 'Heteroskedasticity' means that the larger intensities tend to have larger variations, a common observation with many types of microarray data. The 'heteroskedasticity' nature of the relationship between the mean bead intensities *μ*_*total *_and the variation in bead intensities *σ*_*wtrep *_is apparent (in red in Figure 1). On the other hand, a trend of growth in the standard deviation of mean estimates *σ*_*μ *_with increase of mean intensities across the dynamic range is not certain (in blue in Figure 1) and even *σ*_*μ *_= *const *cannot be excluded (the case of purely systematic error). Given that the number of technical replicates *K *is only four, obtaining good estimates for *σ*_*μ *_especially in the higher intensities region is impossible. As such, we strongly advocate the use of equation (5), which only relies on *σ*_*wtrep *_for computing the correct summary statistic, instead of equation (2).

### MT1-MMP data: proof for cell cycle pathway involvement

Golubkov et al. [7] published the expression profiles of mammary epithelial cells without and after transfection with a plasmid carrying the membrane type-1 matrix metalloproteinase (MT1-MMT) gene recorded with the Illumina platform. Originally, the expression data was first normalized using the "normalize.quantiles" [16] routine of Bioconductor and then imported into GeneSpring for Welch's t-test (thus, using *σ*_*μ *_as the summary statistic). A total of 207 differentially expressed genes were determined with cutoff criteria of p ≤ 0.05 and absolute fold change (FC) of at least 2.

In this work, the original expression data was first normalized (see Array normalization procedure section) prior to statistical treatment. Welch's t test was then performed for both *σ*_*μ *_and *σ*_*wtrep*_, which yielded 215 and 218 differentially expressed genes respectively upon applying the same cutoff criteria. For the three lists consisting of 207, 215 and 218 gene candidates, RefSeq IDs were extracted. The resulting 202, 200 and 203 RefSeq IDs were then separately submitted to NIH DAVID [17] for KEGG pathway mapping. Furthermore, 19815 RefSeq IDs were extracted from the Illumina Human-6 Expression BeadChip annotation file and submitted to DAVID as the background list.

With reference to Table 3, only KEGG pathways with EASE[18] score ≤ 0.05 and count ≥ 5 are shown. In a nutshell, the EASE score is a P- value of a more conservative version of the Fisher's exact test while count denotes the number of genes in the differentially expressed gene list that belongs to a particular pathway. Notably, our analysis with the statistic *σ*_*μ *_essentially repeats the outcome of the work by Golubkov et al. with respect to the pathways (Table 3) and regulated genes (the overlap between the two lists includes 176 genes out of 200 and 202 genes respectively); thus, the differences in the normalization had a small effect.

**Table 3:** KEGG pathways elucidated from the MT1-MMP data.

**KEGG pathway**	*σ*_*μ*_ **[7]**	*σ*_*μ*_	*σ*_*wtrep*_
HSA01430 : Cell communication	✓	✓	✓
HSA04540 : Gap junction	✓	✓	✓
HSA04610 : Complement and coagulation cascades	✓	✓	✓
HSA04110 : Cell cycle			✓

The first column indicates the name of the KEGG pathways. Only KEGG pathways with EASE score ≤ 0.05 and count ≥ 5 are included. The second to fourth columns indicate KEGG pathway found by Golubkov V.S. et. al. [7], *σ*_*μ *_and *σ*_*wtrep *_respectively. Cutoff criteria of EASE score ≤ 0.05 and count ≥ 5 was applied.

Application of the statistic *σ*_*wtrep *_dramatically influences the result. Suddenly, additional cell cycle genes are significantly regulated in transfected cells and the cell cycle pathway pops up in the DAVID analysis. Table 4 highlights the two genes out of a total of six significantly up-regulated genes from the elucidated cell cycle pathway. These two genes, Cyclin A1 and CDC45L, are found by *σ*_*wtrep *_but not by *σ*_*μ*_. Consequently, the addition of these two genes resulted in an improved EASE score of 0.04 (from 0.29). The elucidation of this pathway has substantiated the authors' claim with statistically significant expression arguments that the cell cycle is disrupted with observable mitotic spindle aberrations and aneuploidy in the 184B5-MT cells [7].

**Table 4:** Cell cycle genes in the MT1-MMP data.

		*σ*_*μ *_**[7]**		*σ*_*wtrep*_		
			
**Gene Symbol**	**RefSeq ID**	**log**_**2 **_**FC**	**p value**	**log**_**2 **_**FC**	**p value**	**Gene Description**
CCNA1	NM_003914	-	≥ 0.05	1.12	0.00	Cyclin A1
CDC45L	NM_003504	-	≥ 0.05	1.05	0.00	CDC45 cell division cycle 45-like

CCNB1	NM_031966	1.30	< 0.05	1.44	0.00	Cyclin B1
CCNB2	NM_004701	1.27	< 0.05	1.39	0.00	Cyclin B2
CDC2	NM_033379	1.10	< 0.05	1.20	0.00	cell division cycle 2, G1 to S and G2 to M
CDC20	NM_001255	2.02	< 0.05	2.01	0.00	Cell division cycle 20 homolog

The first, second and last column indicate the gene symbol, the RefSeq ID and the description of each cell cycle gene respectively. The third and fourth columns indicate the logarithm fold change and the P-value of each gene found by Golubkov V.S. et. al. [7], which is analogous to using *σ*_*μ *_as the summary statistic. The fifth and sixth columns indicate the logarithm fold change and the P-value of each gene found by using *σ*_*wtrep *_as the summary statistic. Cutoff criteria of p ≤ 0.05 and |FC| ≥ 2 were applied. The genes in the upper section were found only by the *σ*_*wtrep *_statistic, whereas the genes in the lower section were detected with significance by both statistics.

### Human spermatogenesis data: proof for the N-glycan, the tight and the adherens junction pathway involvement

Platts et al. [8] studied RNA expression in ejaculates of normal and zoospermic men both with the Affymetrix and the Illumina platforms. The Affymetrix expression data of 13 normal and 8 teratozoospermic men was processed by the MBEI (PM-MM) algorithm after invariant set normalization to obtain the gene expression values using the DChip software [19]. The Illumina BeadChip study included only 5 out of the 13 normal but all zoospermic examples. The authors used the same procedure for elucidating differentially expressed genes in both cases [8].

In this work, 5 out of the 13 normal and the 8 teratozoospermic samples from the Affymetrix experiment that were used by Platts et al. in their Illumina experiment (N1, N5, N6, N11, N12) were re-analyzed. The gene-level data was normalized (see Materials and methods), followed by a pair-wise t-test with *ν *and *ω *as the summary statistic (equations 6 and 7). This resulted in a total of 11932 differentially expressed genes (6861 RefSeq IDs) after applying cutoff criteria of p ≤ 0.01 and |FC| ≥ 2. In a similar fashion, the expression data from the corresponding 5 normal and 8 teratozoospermic of the Illumina experiment was normalized and statistically treated for both *σ*_*μ *_and *σ*_*wtrep*_. Using the same cutoff criteria, the two analyses yielded 2464 DEGs (2109 RefSeq IDs) and 4149 DEGs (3316 RefSeq IDs) respectively. Since the number of differentially expressed genes for *σ*_*wtrep *_is increased for the same cutoff criteria, this statistic exhibited a higher statistical power.

The three RefSeq ID lists were submitted to DAVID for KEGG pathway mapping. For Affymetrix, the background list was set to a list of 39647 ReqSeq IDs that was extracted from the HG-U133 (version 2) annotation file. For Illumina, the same list of 19815 RefSeq IDs from MT1-MMP example (see previous section) was submitted as the background.

Analogous to Table 3, only KEGG pathways with EASE score ≤ 0.05 and count ≥ 5 are shown in Table 5. The elucidation of the proteosome and ubiquitin mediated proteolysis pathways by the re-analyzed Affymetrix expression data is consistent with the authors' finding that there is a severe suppression of the proteosomal RNAs associated with the ubiquitin-proteasomal pathway (UPP) in the teratozoospermic samples. On the other hand, the Illumina analysis revealed the proteosome but not the ubiquitin mediated proteolysis pathway. Even though the count for the ubiquitin-mediated proteolysis pathway had increased from 11 to 15 when *σ*_*wtrep *_was used instead of *σ*_*μ*_, this increase only improved the EASE score from 0.07 to 0.066. This marginally missed the significance cutoff of ≤ 0.05. But more interestingly, the Illumina analysis was able to elucidate a few pathways involved in spermatogenesis [20], like the N-glycan biosynthesis [21-23], adherens and tight junction [24-26] when *σ*_*wtrep *_was used as the summary statistic.

**Table 5:** KEGG pathways elucidated from the human spermatogenesis data.

**KEGG pathway**	**Affymetrix**	**Illumina**	
	
	*ω*	*σ*_*wtrep*_	*σ*_*μ*_
HSA00190 : Oxidative phosphorylation	✓	✓	✓
HSA00970 : Aminoacyl-tRNA synthetases	✓	✓	✓
HSA03010 : Ribosome	✓	✓	✓
HSA03050 : Proteosome	✓	✓	✓
HSA00010 : Glycolysis/Gluconeogenesis		✓	✓
HSA00030 : Pentose phosphate pathway		✓	✓
HSA00193 : ATP synthesis		✓	✓
HSA00530 : Aminosugars metabolism		✓	✓
HSA00640 : Propanoate metabolism		✓	✓
HSA03020 : RNA polymerase		✓	✓
HSA03060 : Protein export		✓	✓
HSA04110 : Cell cycle		✓	✓
MMU03010 : Ribosome	✓	✓	
HSA04120 : Ubiquitin mediated proteolysis	✓		
HSA00020 : Citrate cycle (TCA cycle)	✓		
HSA00240 : Pyrimidine metabolism		✓	
HSA00251 : Glutamate metabolism		✓	
HSA00510 : N-glycan biosynthesis		✓	
HSA03022 : Basal transcription factors		✓	
HSA04520 : Adherens junction		✓	
HSA04530 : Tight junction		✓	
HSA00620 : Pyruvate metabolism			✓

The first column indicates the name of the KEGG pathways. Only KEGG pathways with EASE score ≤ 0.05 and count ≥ 5 are included. The second column indicates KEGG pathways found by Affymetrix. The third and fourth columns indicate the KEGG pathways found by using *σ*_*μ *_and *σ*_*wtrep *_respectively. Cutoff criteria of EASE score ≤ 0.05 and count ≥ 5 were applied.

Table 6 highlights 8 out of 17 genes from the elucidated N-glycan pathway that are found by *σ*_*wtrep *_but not *σ*_*μ*_. Using *σ*_*wtrep *_as the summary statistic, the P-values for these 8 genes were improved. With the addition of these 8 genes, the EASE score improved from 0.143 to 0.003. More notably, genes with biological evidence on the role of N-glycan biosynthesis pathway in spermatogenesis begin to surface with the application of the correct summary statistic. There is independent experimental evidence that proves the involvement of the detected genes. For example, beta-1,4-galactosyltransferase-I (B4GALT1) is found to bind with ZP3 receptors on the sperm surface [27]. Also, there is a reported increase in dehydrodolichyl diphosphate synthase (DHDDS) activity in prepuberal rats during early stages of spermatogenesis [28,29]. MAN2A2 is also found to be implicated in male infertility of the alpha-mannosidase IIx (MX) gene knockout mouse [21-23].

**Table 6:** N-glycan biosynthesis genes in human spermatogenesis data.

		*σ*_*μ*_		*σ*_*wtrep*_		
			
**Gene Symbol**	**RefSeq ID**	**log**_**2 **_**FC**	**p value**	**log**_**2 **_**FC**	**p value**	**Gene Description**
B4GALT1	NM_001497	-0.45	0.499	-1.08	0.000	UDP-Gal:betaGlcNAc beta 1,4- galactosyltransferase, polypeptide 1
DDOST	NM_005216	-2.22	0.067	-1.18	0.000	dolichyl-diphosphooligosaccharide-protein glycosyltransferase
DHDDS	NM_024887	3.60	0.049	3.62	0.000	dehydrodolichyl diphosphate synthase
DPM1	NM_003859	-0.65	0.320	-1.06	0.000	dolichyl-phosphate mannosyltransferase polypeptide 1, catalytic subunit
GANAB	NM_198334	1.45	0.045	2.12	0.000	glucosidase, alpha; neutral AB
MAN1A2	NM_006699	-1.27	0.019	-1.28	0.000	mannosidase, alpha, class 1A, member 2
MAN2A2	NM_006122	-0.81	0.001	-1.06	0.000	mannosidase, alpha, class 2A, member 2
UGCGL2	NM_020121	-0.97	0.003	-1.25	0.000	UDP-glucose ceramide glucosyltransferase-like 2

ALG2	NM_033087	-2.18	0.000	-2.20	0.000	asparagine-linked glycosylation 2 homolog (S. cerevisiae, alpha-1,3-mannosyltransferase)
ALG5	NM_013338	-1.90	0.000	-1.71	0.000	asparagine-linked glycosylation 5 homolog (S. cerevisiae, dolichyl-phosphate beta-glucosyltransferase)
ALG8	NM_024079	-1.36	0.001	-1.52	0.000	asparagine-linked glycosylation 8 homolog (S. cerevisiae, alpha-1,3-glucosyltransferase)
B4GALT2	NM_003780	2.33	0.006	2.29	0.000	UDP-Gal:betaGlcNAc beta 1,4- galactosyltransferase, polypeptide 2
MAN2A1	NM_002372	-1.21	0.055	-1.33	0.000	mannosidase, alpha, class 2A, member 1
MGAT4A	NM_012214	-1.84	0.001	-1.72	0.000	mannosyl (alpha-1,3-)-glycoprotein beta-1,4-N-acetylglucosaminyltransferase, isozyme A
OGT	NM_181673	-2.31	0.009	-2.12	0.000	O-linked N-acetylglucosamine (GlcNAc) transferase (UDP-N-acetylglucosamine:polypeptide-N-acetylglucosaminyl transferase)
RPN1	NM_002950	-1.46	0.001	-1.51	0.000	ribophorin I
RPN2	NM_002951	-1.95	0.000	-2.00	0.000	ribophorin II

The first, second and last column indicate the gene symbol, the RefSeq ID and the description of each N-glycan biosynthesis gene respectively. The third and fourth columns indicate the logarithm fold change and the P-value of each gene found by using *σ*_*μ *_as the summary statistic. The fifth and sixth columns indicate the logarithm fold change and the P-value of each gene found by using *σ*_*wtrep *_as the summary statistic. The cutoff criteria p ≤ 0.01 and |FC| ≥ 2 were applied. The first 8 genes are found to be statistically significant by *σ*_*wtrep *_only, the rest was found with both statistics.

In the case of the tight junction pathway, 17 out of 37 genes from this elucidated pathway are found by *σ*_*wtrep *_but not *σ*_*μ *_(Table 7). The improvement in the P-values of these additional 17 genes effected a marked improvement in EASE score from 0.206 to 0.012. For the adherens junction pathway, 14 out of 26 genes are found only by *σ*_*wtrep *_(Table 8). Consequently, the EASE score improved dramatically from 0.527 to 0.037. As a result of applying the correct summary statistic, the genes CLDN1, CSNK2A2, CTNNA1, JAM3, and TJP1 have surfaced from the analysis. Claudin-1 (CLDN1) is involved in the developmental regulation of the tight junctions in mouse testis [30], while casein kinase 2, alpha prime polypeptide (CSNK2A2) null male mice are infertile and their cells from spermatogonia to early spermatids suffer nuclear envelope protrusions, outer membrane swelling and inner membrane disruption [31]. Also, the assembly of adherens junctions between Sertoli and germ cells was associated with a transient induction in the steady-state mRNA and protein levels of cadherins and catenins. In particular, alpha-catenin (CTNNA1) expression was seen in semi-quantitative reverse transcription polymerase chain reaction and immunoblotting [32]. Furthermore, the expression of zona occludens 1 (TJP1) in rats is regulated in vitro during the assembly of inter-Sertoli tight junctions during spermatogenesis [33] while JAM-3 is a protein required for spermatid differentiation [34].

**Table 7:** Tight junction genes in human spermatogenesis data.

		*σ*_*μ*_		*σ*_*wtrep*_		
			
**Gene Symbol**	**RefSeq ID**	**log**_**2 **_**FC**	**P-value**	**log**_**2 **_**FC**	**P-value**	**Gene Description**
ACTG1	NM_001614	-0.81	0.064	-1.14	0.000	actin, gamma 1
CLDN1	NM_021101	-2.47	0.059	-1.63	0.000	claudin 1
CLDN16	NM_006580	-1.20	0.019	-1.55	0.000	claudin 16
CLDN5	NM_003277	1.15	0.286	1.42	0.000	claudin 5 (transmembrane protein deleted in velocardiofacial syndrome)
CLDN6	NM_021195	2.48	0.135	1.88	0.000	claudin 6
CSNK2A2	NM_001896	-1.00	0.014	-1.57	0.000	casein kinase 2, alpha prime polypeptide
CTNNA1	NM_001903	-0.93	0.009	-1.04	0.000	catenin (cadherin-associated protein), alpha 1, 102 kDa
EXOC3	NM_007277	-1.26	0.019	-1.43	0.000	exocyst complex component 3
EXOC4	NM_021807	-0.78	0.012	-1.04	0.000	exocyst complex component 4
GNAI2	NM_002070	1.02	0.110	1.52	0.000	guanine nucleotide binding protein (G protein), alpha inhibiting activity polypeptide 2
JAM3	NM_032801	-0.86	0.005	-1.07	0.000	junctional adhesion molecule 3
KRAS	NM_004985	-2.85	0.032	-2.43	0.000	v-Ki-ras2 Kirsten rat sarcoma viral oncogene homolog
MYH9	NM_002473	1.09	0.183	1.02	0.000	myosin, heavy chain 9, non-muscle
PPP2R2B	NM_181676	-1.22	0.020	-1.97	0.000	protein phosphatase 2 (formerly 2A), regulatory subunit B, beta isoform
PPP2R3A	NM_002718	1.42	0.013	1.50	0.000	protein phosphatase 2 (formerly 2A), regulatory subunit B", alpha
RAB13	NM_002870	-1.17	0.118	-1.68	0.000	RAB13, member RAS oncogene family
TJP1	NM_175610	-2.63	0.038	-2.10	0.000	tight junction protein 1 (zona occludens 1)

AKT3	NM_181690	-1.16	0.001	-1.16	0.000	v-akt murine thymoma viral oncogene homolog 3 (protein kinase B, gamma)
CDC42	NM_044472	-1.67	0.001	-1.51	0.000	cell division cycle 42 (GTP binding protein, 25 kDa)
CLDN11	NM_005602	-2.03	0.000	-2.06	0.000	claudin 11 (oligodendrocyte transmembrane protein)
CLDN14	NM_012130	2.92	0.006	2.90	0.000	claudin 14
CSDA	NM_003651	-2.28	0.000	-2.48	0.000	cold shock domain protein A
CSNK2B	NM_001320	-2.61	0.006	-2.69	0.000	casein kinase 2, beta polypeptide
CTNNA2	NM_004389	-1.64	0.002	-1.88	0.000	catenin (cadherin-associated protein), alpha 2
CTTN	NM_138565	-1.91	0.008	-2.05	0.000	cortactin
EPB41L3	NM_012307	-1.85	0.001	-1.71	0.000	erythrocyte membrane protein band 4.1-like 3
MYH10	NM_005964	-1.34	0.000	-1.35	0.000	myosin, heavy chain 10, non-muscle
MYL6	NM_079423	-1.62	0.000	-1.78	0.000	myosin, light chain 6, alkali, smooth muscle and non-muscle
PPP2CA	NM_002715	-2.69	0.002	-2.40	0.000	protein phosphatase 2 (formerly 2A), catalytic subunit, alpha isoform
PPP2CB	NM_004156	-1.43	0.005	-1.83	0.000	protein phosphatase 2 (formerly 2A), catalytic subunit, beta isoform
PPP2R1B	NM_181699	-2.14	0.003	-2.34	0.000	protein phosphatase 2 (formerly 2A), regulatory subunit A, beta isoform
PPP2R1B	NM_181699	-1.47	0.011	-1.18	0.000	protein phosphatase 2 (formerly 2A), regulatory subunit A, beta isoform
PPP2R2A	NM_002717	-1.53	0.002	-1.86	0.000	protein phosphatase 2 (formerly 2A), regulatory subunit B, alpha isoform
PPP2R2B	NM_181677	2.51	0.007	2.29	0.000	protein phosphatase 2 (formerly 2A), regulatory subunit B, beta isoform
PRKCH	NM_006255	-1.20	0.002	-1.03	0.000	protein kinase C, eta
PTEN	NM_000314	-2.30	0.004	-1.61	0.000	phosphatase and tensin homolog (mutated in multiple advanced cancers 1)
RHOA	NM_001664	-1.81	0.001	-1.58	0.000	ras homolog gene family, member A

CTNNA3	NM_013266	-1.03	0.007	-0.92	0.000	catenin (cadherin-associated protein), alpha 3

The first, second and last column indicate the gene symbol, the RefSeq ID and the description of each Tight junction gene respectively. The third and fourth columns indicate the logarithm fold change and the P-value of each gene found by using *σ*_*μ *_as the summary statistic. The fifth and sixth columns indicate the logarithm fold change and the P-value of each gene found by using *σ*_*wtrep *_as the summary statistic. A cutoff criteria of p ≤ 0.01 and |FC| ≥ 2 was applied. The first 17 genes are found to be statistically significant by *σ*_*wtrep *_only. The last row contain a gene excluded by *σ*_*wtrep *_due to |FC| ≤ 2 although p ≤ 0.01. The genes upper in the section were found only by the *σ*_*wtrep *_statistic, the remainder was found with both approaches except of the last gene entry recovered only by the *σ*_*μ *_statistic (it was filtered out due the fold change criterion).

**Table 8:** Adherens junction genes in human spermatogenesis data.

		*σ*_*μ*_		*σ*_*wtrep*_		
			
**Gene Symbol**	**RefSeq ID**	**log**_**2 **_**FC**	**P-value**	**log**_**2 **_**FC**	**P-value**	**Gene Description**
ACTG1	NM_001614	-0.81	0.064	-1.14	0.000	actin, gamma 1
BAIAP2	NM_006340	1.32	0.016	1.27	0.000	BAI1-associated protein 2
CREBBP	NM_004380	-0.98	0.002	-1.11	0.000	CREB binding protein (Rubinstein-Taybi syndrome)
CSNK2A2	NM_001896	-1.00	0.014	-1.57	0.000	Casein kinase 2, alpha prime polypeptide
CTNNA1	NM_001903	-0.93	0.009	-1.04	0.000	catenin (cadherin-associated protein), alpha 1, 102 kDa
FER	NM_005246	-1.37	0.015	-1.73	0.000	fer (fps/fes related) tyrosine kinase (phosphoprotein NCP94)
IQGAP1	NM_003870	-2.66	0.026	-2.27	0.000	IQ motif containing GTPase activating protein 1
MAPK1	NM_002745	-1.56	0.014	-1.34	0.000	mitogen-activated protein kinase 1
MAPK3	NM_002746	1.75	0.042	1.88	0.000	mitogen-activated protein kinase 3
PTPRF	NM_130440	-0.91	0.000	-1.10	0.000	protein tyrosine phosphatase, receptor type, F
SMAD2	NM_005901	-1.27	0.027	-1.59	0.000	SMAD family member 2
TCF7	NM_003202	3.13	0.045	2.90	0.000	transcription factor 7 (T-cell specific, HMG-box)
TJP1	NM_175610	-2.63	0.038	-2.10	0.000	tight junction protein 1 (zona occludens 1)
WASL	NM_003941	-1.99	0.047	-1.42	0.000	Wiskott-Aldrich syndrome-like

ACP1	NM_004300	-1.82	0.009	-1.66	0.000	acid phosphatase 1, soluble
ACP1	NM_007099	-1.77	0.000	-1.70	0.000	acid phosphatase 1, soluble
CDC42	NM_044472	-1.67	0.001	-1.51	0.000	cell division cycle 42 (GTP binding protein, 25 kDa)
CSNK2B	NM_001320	-2.61	0.006	-2.69	0.000	casein kinase 2, beta polypeptide
CTNNA2	NM_004389	-1.64	0.002	-1.88	0.000	catenin (cadherin-associated protein), alpha 2
MAP3K7	NM_145333	-1.52	0.000	-1.39	0.000	mitogen-activated protein kinase kinase kinase 7
MAPK1	NM_138957	-1.19	0.005	-1.23	0.000	mitogen-activated protein kinase 1
MAPK1	NM_138957	-1.68	0.002	-2.03	0.000	mitogen-activated protein kinase 1
RHOA	NM_001664	-1.81	0.001	-1.58	0.000	ras homolog gene family, member A
SMAD4	NM_005359	-1.02	0.000	-1.07	0.000	SMAD family member 4
SORBS1	NM_015385	1.86	0.001	1.99	0.000	sorbin and SH3 domain containing 1
WASF3	NM_006646	-1.62	0.002	-1.80	0.000	WAS protein family, member 3

CTNNA3	NM_013266	-1.03	0.007	-0.92	0.000	catenin (cadherin-associated protein), alpha 3

The first, second and last column indicate the gene symbol, the RefSeq ID and the description of each Tight junction gene respectively. The third and fourth columns indicate the logarithm fold change and the P-value of each gene found by using *σ*_*μ *_as the summary statistic. The fifth and sixth columns indicate the logarithm fold change and the P-value of each gene found by using *σ*_*wtrep *_as the summary statistic. Cutoff criteria of p ≤ 0.01 and |FC| ≥ 2 were applied. The first 14 genes are found to be statistically significant by *σ*_*wtrep *_only. The lower part of the table lists genes found by both statistics except for the last row that contains a gene excluded by *σ*_*wtrep *_due to |FC| ≤ 2 (although p ≤ 0.01).

**Figure 2:**
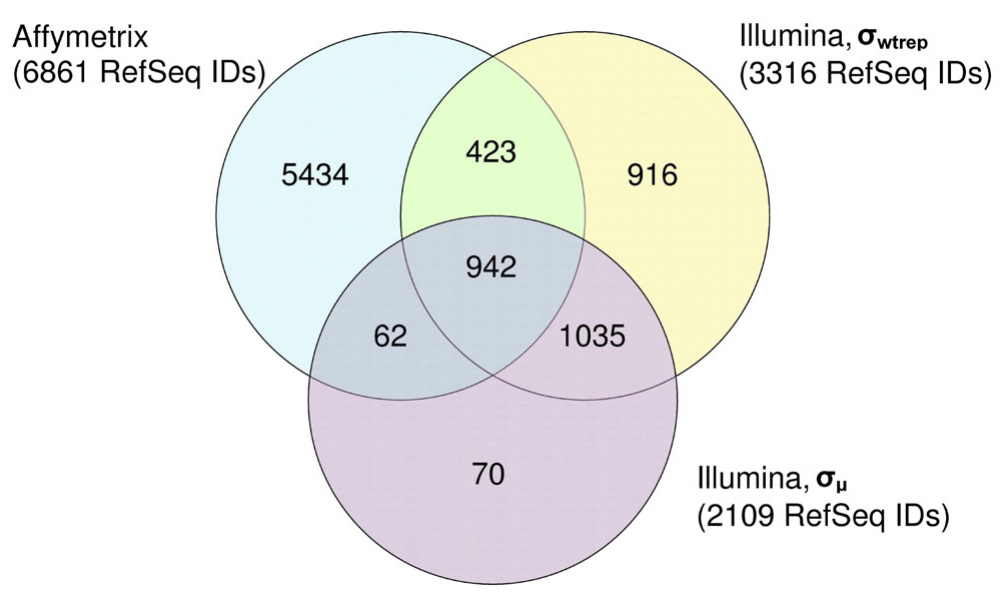
**Venn diagram of gene list overlap**. The Venn diagram of the distribution of differentially expressed genes (based on the cutoff criteria p ≤ 0.01 and |FC| ≥ 2) between Affymetrix and Illumina spermatogenesis datasets is presented.

The concordance between the three RefSeq lists was next investigated. The results are shown in Figure 2. Through the derivation of a parallel summary statistic to the Affymetrix one, an addition of 423 concordance RefSeq IDs was recovered. This is an increase of 45% (423 out of 942) in the concordance region. The correct summary statistic also improved gene discovery. Out of the 916 RefSeq IDs that were unique to the analysis by *σ*_*wtrep*_, 8 (B4GALT1, DHDDS, MAN2A2, CLDN1, CSNK2A2, CTNNA1, JAM3, TJP1) were validated spermatogenesis genes from the N-glycan biosynthesis, tight and adherens junction pathways. These pathways were not reported by Affymetrix. Another interesting observation is that the RefSeq list obtained with *σ*_*μ *_as a statistic almost formed a subset of the list yielded by *σ*_*wtrep*_. In total, 93.7% of its ReqSeq IDs were found within the list generated with *σ*_*wtrep*_. At first glance, it seems that the effect of using *σ*_*μ *_rather than *σ*_*wtrep *_does not seem detrimental. However, the rankings of the P-values in this overlapped region were not preserved, similar to the case of our Illumina spike experiments analysis. As such, the top 100 candidate list for example will be quite different when *σ*_*μ *_instead of *σ*_*wtrep *_is used.

## Conclusion

Due to the specific statistical nature of the Illumina BeadChip summary data as means and standard deviations of subsets of measurements, the typical statistical workflow of finding differentially expressed genes cannot be applied to this data directly. To remedy this situation, *σ*_*wtrep *_is proposed as correct summary statistic of the Illumina BeadChip. Our work has shown that the same Illumina BeadChip data from published experiments churns out better differentially expressed gene selection after applying our proposed summary statistic.

This was particularly evident in the low concentration range of the Illumina spike experiment [11,14]. Given that this range is typical for the endogenous gene expression, the improvement should also be observed in biological experiments as well. Indeed, the superior statistical significance contributed markedly to more successful biological pathway elucidations. This was demonstrated with the MT1-MMP [7] data as well as the human spermatogenesis [8] data. For these two examples, more relevant differentially expressed genes were revealed when our proposed summary statistic was applied. In fact, a number of these genes has already been independently validated in the literature [21,27-34]. Their biological significance was demonstrated through functional studies like gene knock-out, mutagenesis and quantification studies like RT-PCR and immunoblotting. Finally in the context of cross-platform comparison between Affymetrix and Illumina, more concordant results were recovered for the spermatogenesis expression profile [8]. This should not be surprising because our summary statistic is a close parallel to that of Affymetrix.

To conclude, our work is most relevant and imperative to any investigator who wants to derive more accurate differentially expressed gene lists from Illumina data.

## Materials and methods

### The Illumina spike experiment

We exploited the dataset from a published artificial spike experiment [11,14]; the complete dataset was obtained as a personal communication by Semyon Kruglyak [See additional file 1]. In total, 4 versions for each of eight artificial polyadenylated RNAs (bla, cat, cre, e1a, gfp, gst, gus, lux) were generated by the authors. Although it was not mentioned in [11,14], the dataset contains two versions of another artificial polyadenylated RNA (neo). Therefore, there are altogether 34 unique labeled and spiked 50 mers against a human cRNA background for each of the spike concentrations. The pooled spike RNAs were tested at a total of twelve different concentrations (0, 0.01, 0.03, 0.1, 0.3, 1, 3, 10, 30, 100, 300, 1000) pM. Each of the spiked and labeled samples (at 1.5 *μ*g per sample) was hybridized in quadruplicates across 48 arrays on eight different Human-6 Expression BeadChips.

### The MT1-MMT (membrane type-1 matrix metalloproteinase) experiment

This dataset available as NCBI GEO GSE5095 was complemented with replicate-specific standard errors and number of beads in a private communication by Vladislav S. Golubkov. In the experiment, 184B5 human normal mammary epithelial cells were transfected with MT1-MMP [7]. Total RNA was then isolated from the 184B5-MT and 184B5 cell culture, following DNA-chip RNA expression profiling using Illumina Human-6 Expression BeadChips.

### The human spermatogenesis experiment

The published expression profile dataset NCBI GEO GSE6969 from human ejaculates was used [8]. In the experiment, the samples were collected from 17 normal fertile men and 14 teratozoospermic men aged between 21 to 57. Upon RNA isolation of the spermatozoa, RNA expression profiling was carried out on both the Affymetrix and Illumina platform. 4 out of 17 normal and 6 out of 14 teratozoospermic samples are profiled by the Illumina Human-8 Expression BeadChips while the remaining 13 normal and 8 teratozoospermic samples were profiled by the Affymetrix HG-U133 (version 2) GeneChips. In addition, 5 out of these 13 normal samples and the same 8 teratozoospermic samples were profiled again by the Illumina Human-6 Expression BeadChips.

### Array normalization procedure

Our proposed procedure is inspired by quantile normalization and by the scaling method used by Affymetrix [16]. The quantile normalization variant is applied to groups of technical replicates with the goal to achieve equal spread in the distribution of the bead intensities for each array. Then, the scaling method is applied to all the arrays to ensure that the medians of all arrays are equal.

For the sake of simplicity, the normalization procedure illustrated below will be based on only one treatment condition. The same steps will be repeated for other treatment conditions. Therefore, an arbitrary gene *g *from an array consisting of a total of *G *genes with *K *technical replicates each will have summary data *μ*_*g*,*k*_, *σ*_*g*,*k*_, *M*_*g*,*k *_where *g *= 1,...,*G *and *k *= 1,...,*K*.

Log-transformation is first applied on the mean bead intensities for all readings. The *k*-th technical replicate of gene *g *after undergoing log-transformation is depicted as log_2_(*μ*_*g*,*k*_).

First, normalization within the replicate is performed. For the *k*-th technical replicate, the median and standard deviation for the log mean bead intensities within replicate are calculated as

$$\begin{array}{l} median_{\log_{2}(\mu_{k,wtrep})}=median[\log_{2}(\mu_{1,k}),...,\log_{2}(\mu_{G,k})]\\ \sigma_{\log_{2}(\mu_{k,wtrep})}=\sqrt{\frac{1}{G}\sum_{g=1}^{G}{\left[\log_{2}(\mu_{g,k})-median_{\log_{2}(\mu_{k,wtrep})}\right]}^{2}} \end{array}$$

The median-of-medians within replicate and its corresponding standard deviation, hereby depicted as *MOM*_*wtrep *_and $\sigma_{MOM_{wtrep}}$ respectively, are given as

$$\begin{array}{c} \sigma_{MOM_{wtrep}}=\sigma_{\log_{2}(\mu_{k*,wtrep})}\\ \text{where }k*=\underset{k}{\arg}[median_{\log_{2}(\mu_{k,wtrep})}=MOM_{wtrep}] \end{array}$$

Therefore, the normalized log mean bead intensities within replicate for the *k*-th technical replicate of gene *g *is defined as

$$\log_{2}(\mu_{g,k,normwtrep})=\left[\frac{\log(\mu_{g,k})-median_{\log_{2}(\mu_{k,wtrep})}}{\sigma_{\log_{2}(\mu_{k,wtrep})}}\right]*\sigma_{MOM_{wtrep}}+MOM_{wtrep}$$

The median for the normalized log mean bead intensities within the *k*-th replicate is then re-calculated in a similar fashion as before, where

$$median_{\log_{2}(\mu_{k,normwtrep})}=median[\log_{2}(\mu_{1,k,normwtrep}),...,\log_{2}(\mu_{G,k,normwtrep})]$$

The median-of-medians across replicates, depicted as *MOM*_*acrep*_, is defined as

$$MOM_{acrep}=median[median_{\log_{2}(\mu_{1,normwtrep})},...,median_{\log_{2}(\mu_{K,normwtrep})}]$$

Therefore, the normalized log mean bead intensities across replicates will be

$$\log_{2}(\mu_{g,k,normacrep})=\log_{2}(\mu_{g,k,normwtrep})-median_{\log_{2}(\mu_{k,wtrep})}+MOM_{acrep}$$

The normalized log mean bead intensities across replicates are then transformed back to the original scale, where

$$\mu_{g,k,norm}=2^{\log_{2}(\mu_{g,k,normacrep})}$$

and the corresponding standard deviation is now

*σ*_*g*,*k*,*norm *_= *scale*_σ _* *σ*_*g*,*k*_

$$\text{where }scale_{\sigma }=\sqrt{\frac{\mu_{g,k,norm}}{\mu_{g,k}}}$$

Therefore, after undergoing the array normalization procedure, an arbitrary gene *g *from an array consisting of a total of *G *genes with *K *technical replicates each will now have the summary data *μ*_*g*,*k*,*norm*_, *σ*_*g*,*k*,*norm*_, *M*_*g*,*k *_where *g *= 1,..., *G *and *k *= 1,...,*K*.

Assume that one treatment condition is replicated *K *times in the Beadchip, i.e., *k *= 1,...,*K*. When the mean estimates *μ*_*g*,*k *_are being treated as the intensity values *x*_*g*,*k*,1_, the mean and the incorrect summary statistic of the variance of this treatment condition are given as

$$\begin{array}{c} \mu_{g}=\frac{1}{K}\sum_{i=1}^{K}\mu_{g,k,norm}\\ \begin{array}{ccc} \sigma_{g}^{2}=\frac{1}{K}\sum_{i=1}^{K}{\left(\mu_{g,k}-\mu_{g}\right)}^{2} & \text{where} & n_{g}=K \end{array} \end{array}$$

Note that $\sigma_{g}^{2}$ is equivalent to equation (3).

On the other hand, when no misinterpretation of the summary statistic has occurred, the mean and variance are given as

*μ*_*g *_= *median*(*μ*_*g*,1,*norm*_,...,*μ*_*g*,*K*,*norm*_)

$$\sigma_{g}^{2}=\sigma_{g,k*,norm}^{2}$$

*n*_*g *_= *M*_*g*,k*_

$$\text{where }k*=\underset{k}{\arg}[\mu_{g,k,norm}=\mu_{g}]$$

Note that $\sigma_{g}^{2}$ is now equivalent to equation (5).

### Statistical test procedure

A pair-wise t-test [35] infers if differences exist between the two populations sampled. Furthermore, given 2 treatment conditions *c*1 and *c*2, an arbitrary gene *g *will have two pair of readings *μ*_*g*,*c*1_, *σ*_*g*,*c*1_, *n*_*g*,*c*1 _and *μ*_*g*,*c*2_, *σ*_*g*,*c*2_, *n*_*g*,*c*2_. Then if the two treated samples came from normal populations and if both have equal variances, then the t value for the difference between the two treatment conditions is given as

$$t=\frac{\mu_{g,c2}-\mu_{g,c1}}{s_{\mu_{g,c2}-\mu_{g,c1}}}$$

where $s_{\mu_{g,c1}-\mu_{g,c2}}=\sqrt{\frac{s_{p}^{2}}{n_{g,c1}}+\frac{s_{p}^{2}}{n_{g,c2}}}$, $s_{p}^{2}=\frac{(n_{g,c1}-1)\sigma_{g,c1}^{2}+(n_{g,c2}-1)\sigma_{g,c2}^{2}}{n_{g,c1}+n_{g,c2}-2}$ and the degree of freedom is given as *ν *= *n*_*g*,*c*1 _+ *n*_*g*,*c*2_-2. However, if the two treatment samples do not have equal variances, the t value is given as

$$t=\frac{\mu_{g,c2}-\mu_{g,c1}}{\sqrt{\frac{s_{\mu_{g,c1}}^{2}}{n_{g,c1}}+\frac{s_{\mu_{g,c2}}^{2}}{n_{g,c2}}}}$$

The degree of freedom is given as $v=\frac{{\left(\frac{s_{\mu_{g,c1}}^{2}}{n_{g,c1}}+\frac{s_{\mu_{g,c2}}^{2}}{n_{g,c2}}\right)}^{2}}{\frac{{\left(\frac{s_{\mu_{g,c1}}^{2}}{n_{g,c1}}\right)}^{2}}{n_{g,c1}-3}+\frac{{\left(\frac{s_{\mu_{g,c2}}^{2}}{n_{g,c2}}\right)}^{2}}{n_{g,c2}-3}}$.

This is known as the Welch's t test. For a 2-sided alternate hypothesis *H*_*A *_: *μ*_*g*,*c*1 _≠ *μ*_*g*,*c*2_, reject *H*_0 _: *μ*_*g*,*c*1 _= *μ*_*g*,*c*2 _if |*t*|≥ *t*_*α*(2),*ν*_. It should be noted that the array normalization procedure has been applied before the statistical treatment.

## List of abbreviations

FP: false positive; MT1-MMT: membrane type-1 matrix metalloproteinase; TP: true positive.

## Competing interests

The authors declare that they have no competing interests.

## Authors' contributions

WCW and FE derived the proof for the new summary statistic, designed the study and evaluated the results. WCW carried out the programming. ML reproduced the work to validate the results. ML also corresponded with the respective authors of the study datasets for all additional information. All authors participated in drafting the manuscript and approved the final version.

## Reviewers' comments

### Reviewer's report 1

I. King Jordan, School of Biology, Georgia Institute of Technology

Wong, Loh and Eisenhaber present a novel statistical method for evaluating gene expression data produced using the Illumina BeadChip technology. The fundamental insight that led to the new statistical method is their appreciation that Affymetrix GeneChip microarrays produce single gene expression measurements, while use of Illumina BeadChips yields mean expression values from dozens of identical probes. Therefore, Illumina BeadChip data must be treated differently. Specifically, when technical replicates are available, the standard deviations across replicates for Illumina BeadChip data are best computed as weighted means of the square of the standard deviations of individual measures. In other words, the standard deviations for data sets with technical replicates should be computed from standard errors of individual Illumina BeadChip measures. When this adjustment is applied to several test data sets, the performance of the Illumina BeadChips improves markedly.

While I am not qualified to evaluate the statistical details of their method, the results of its application to the three test data sets appear to be quite convincing. As such, this work represents an important technical development with direct relevance to any study that uses Illumina BeadChip technology.

One of the measures used by the authors to indicate the success of their statistic is increased concordance between lists of differentially expressed genes uncovered by Illumina BeadChip and Affymetrix GeneChip experiments on a spermatogenesis dataset. However, it would seem that the increased replicates of the Illumina BeadChip technology provides for an inherent advantage over the single-measure technology employed with Affymetrix GeneChips. If this is indeed the case, then one may expect improved performance for the Illumina platform relative to Affymetrix and not merely increased concordance as was demonstrated for the spermatogenesis dataset. Do the authors have any sense, or evidence, as to whether the increased sampling of Illumina provides more resolution than Affymetrix? For instance, are the new pathways identified by the Illumina BeadChip analysis of the spermatogenesis dataset a function of the superiority of the platform? Or are the methods complementary, *i.e*. does the Affymetrix analysis uncover pathways missed by Illumina irrespective of the use of the statistical innovations introduced herein?

#### Authors' response

*There is no reason to assume a superiority of either platform given the same quality of probe sequence design. For example, one might imagine several Affymetrix chips to be mounted on the same glass slide and to be hybridized simultaneously (to resemble the situation of several beads per array). In this case, both platforms can be used in the mode of exclusion of the batch-specific constant shift error as described in the text*.

I am wondering about the availability of their method. The authors conclude that the work is relevant, even imperative, to any investigator looking for differentially expressed genes in Illumina data? How are those investigators to use this method – on a web server, as a BioPerl object, as an R routine?

#### Authors' response

*Presently, our code is implemented in Matlab and be obtained on request. It would be straightforward to implement an R version of it so that it can tie back to the bioconductor package in R. Nevertheless, it should not be difficult for any scientist in the area to modify their existing workflow similar to ours based on the equations presented in this paper just by using σ_*wtrep *_as standard deviation*.

### Reviewer's report 2

Mark J. Dunning, Computational Biology Group, Department of Oncology, University of Cambridge, Cancer Research UK Cambridge Research Institute

In my opinion, Wong et. al is a useful addition to the topic of analysis of Illumina data. Whilst the number of publications using Illumina data are growing rapidly, very few authors have tackled the issue of how such data should be analysed. Wong et al. do a very good job of explaining why the usual statistical tests, such as applied to Affymetrix may not be appropriate for Illumina data and that the extra information provided with an Illumina experiment (i.e. accurate gene-specific variances) can produce a more powerful test. It is especially pleasing to see that they are able to pick up biologically relevant results using the new summary statistic.

The investigation into the performance of *σ*_*wtrep *_is well presented. However, a detail in the re-analysis of Golubkov et al. seems to be missing. In the original paper, genes were filtered using the detection scores obtained from Illumina's software. It does not seem that these scores were supplied in GEO, so were these scores also available to Wong et al. as part of their re-analysis? If not, how did they go about reproducing the filtering performed in Golubkov et al.?

#### Authors' response

*Indeed, the data stored in GEO is insufficient to carry out the calculations both in the paper of Golubkov et al. and in this work. We received the standard errors and number of beads through a private communication from Golubkov et al. Our first re-analysis aimed at repeating the work of Golubkov et al. differed from their approach in two aspects. On the one hand, we had another normalization algorithm (see Methods section); on the other hand, we did not carry out filtering. Just to note, the detection score P is calculated from Z-scores of intensities shifted by the background (intensity of negative control spots) and scaled with its standard deviation. In pairwise comparisons involving the Welch's test using the wrong summary statistic σ_*μ*_, the differences of intensities do not depend on their previous correction by a constant background. Regardless of these two differences in our re-analysis, the results are essentially identical to the case of Golubkov et al.: The cell cycle pathway did not appear as significantly regulated*.

The results supplied in the paper were enough to convince me that the summary statistic *σ*_*wtrep *_is better than current alternatives. However, I'm afraid I was a bit unsure of the connection between *σ*_*wtrep *_and the normalization method proposed by the authors. Can I still use *σ*_*wtrep *_in my differential expression analysis if I use the usual quantile normalization?

#### Authors' response

*The summary statistic σ*_*wtrep *_can be used with the usual quantile normalization or any normalization methods. One only has to ensure that the standard deviation *σ*_*k *_of the corresponding *μ*_*k *_be adjusted by a transformation factor i.e. *σ*_*k*(*normalized*) _= A*σ*_*k *_*where *$A=\frac{\mu_{k(normlized)}}{\mu_{k}}$. *After which*, *σ*_*wtrep *_*is computed using equation (4)*.

What motivated the authors to propose this method of normalization? However, I feel that the description of the normalization procedure was not that easy to follow and would benefit from a small worked example if possible. Do the authors plan to make any of the methodology presented in the paper available in open-source software?

#### Authors' response

*In a typical Illumina BeadChip experiment, different treatment conditions should be hybridized within a chip, while their corresponding technical replicates should be distributed across chips. The treatment conditions within a chip shall be exposed to similar systematic and random error. Hence, the differences in spreads among the arrays or treatment conditions should ideally reflect true biological differences. The motivation of our normalization method is to create a two-step normalization procedure whereby the first step forces the same median and spread only among technical replicates while the second step simply ensures that the medians across all the arrays are common. As such, the spreads among the various treatment conditions need not be the same, thus preserving true differences. The software (as a Matlab program) is available on request*.

Aside from these questions, and suggestions to improve readability supplied separately to the authors, I am happy for this manuscript to be published.

Specific Comments for the authors:

• Bottom of Page 2: "But instead of delivering the individual bead intensities, the mean and standard error (i.e. the standard deviation divided by the square root of the number of beads) of the bead intensities, known as the summary data, are reported."

This statement is possibly a bit misleading as the individual bead intensities are available with appropriate scanner modifications (see Dunning et. al). I suggest this statement be changed to acknowledge this, although the summary data are usually the starting points for analysis when using Illumina's software.

• Page 3 paragraph 3 – "Furthermore, this summary statistic will..." This should be changed to either "this summary statistic" or "these summary statistic". I suggest the manuscript be checked for other similar errors.

• Equation 1 should have the sum going from k = 1..K rather than i = 1..K

• Page 5 Paragraph 1 – The weights used in Dunning et. al are the inverse of $\sigma_{k}^{2}$ rather than *σ*_*k*_.

• Page 5 Paragraph 3 -"Due to the multiple copies of the same probe within a single Illumina array, the standard variation can be computed...."

Should be standard deviation rather than standard variation?

• Page 6 Paragraph 4 – "On the other hand, a trend of growth of mean estimates *σ*_*μ*_.."

Should this be standard deviation of mean estimates?

#### Authors' response

*The suggested amendments have been made accordingly*.

### Reviewer's report 3

Shamil Sunyaev, Division of Genetics, Dept. of Medicine, Brigham & Women's Hospital and Harvard Medical School

This manuscript describes a new statistical method for the analysis of Illumina BeadChip microarrays. The authors realized that variance is underestimated for these microarrays because the measurements themselves are averaged over multiple probes. Thus, they suggest a new estimate of variance to be used in the analysis based on the Welch's t-test.

The manuscript is well written. The statistical approach is straightforward but has been convincingly demonstrated to produce biologically meaningful results. The authors show that the corrected standard deviation estimate helps obtaining better results for the spike dataset (Chudin et al.) and also reveal more biologically relevant genes in the human spermatogenesis dataset and mammary epithelial dataset.

As an outsider in this field, I do not understand why the analysis is based on the t-test, which heavily depends on sample estimates of variance and assumes normality. It seems that non-parametric method may suite the problem better.

#### Authors' response

*First of all, it is important to note that the summary data is computed using the arithmetic mean and the standard deviation formulas. These formulas are the maximum likelihood estimates (MLEs) of the normal distribution. In other words, normality is innately assumed on the summary data. Furthermore, the typical sample size for each gene measurement in Illumina is about 30 and t-test is known to be robust when sample size is large. More notably, t-test is robust against assumption violations as long as the sample sizes are almost equal and that only two-tailed hypotheses are considered. These were the conditions for all our test cases*.

## Appendix 1

**Proof for **$\sigma_{total}^{2}=\sigma_{wtrep}^{2}+\sigma_{\mu }^{2}$

With reference to Table 1, if the individual bead intensities are available, the mean *μ*_*total *_and squared standard deviation $\sigma_{total}^{2}$ of the total set of bead intensities across *K *technical replicates can be computed as

(8) $$\mu_{total}=C\cdot \sum_{i=1}^{K}\sum_{n=1}^{M_{k}}x_{k,n}$$

(9) $$\sigma_{total}^{2}=C\cdot \sum_{i=1}^{K}\sum_{n=1}^{M_{k}}x_{k,n}^{2}-\mu_{total}^{2}$$

where, for convenience of notation, we introduce

(10) $$C={\left(\sum_{k=1}^{K}M_{k}\right)}^{-1}$$

On the other hand, only the summary data are given. Then the mean *μ*_*total *_and the squared standard deviation $\sigma_{\mu }^{2}$ of the average bead intensities across *K *technical replicates are given as

(11) $$\mu =C\sum_{i=1}^{K}M_{k}\mu_{k}=C\sum_{i=1}^{K}\sum_{n=1}^{M_{k}}x_{k,n}$$

(12) $$\sigma_{\mu }^{2}=C\sum_{k=1}^{K}M_{k}\mu_{k}^{2}-{\left[C\sum_{k=1}^{K}M_{k}\mu_{k}\right]}^{2}$$

Clearly, the weighted average of the *K *bead intensity averages is equal to *μ*_*total *_(equation 8 and 11). For later usage, the equation (12) can be transformed by representing *μ*_*k *_by the average of actual bead intensities $M_{k}^{-1}\sum_{n=1}^{M_{k}}x_{k,n}$.

(13) $$\sigma_{\mu }^{2}=C\cdot \sum_{k=1}^{K}M_{k}^{-1}{\left(\sum_{n=1}^{M_{k}}x_{k,n}\right)}^{2}-C^{2}{\left(\sum_{k=1}^{K}\sum_{n=1}^{M_{k}}x_{k,n}\right)}^{2}$$

On the other hand, the variance in bead intensities across *K *technical replicates $\sigma_{wtrep}^{2}$ is defined as weighted average

(14) $$\sigma_{wtrep}^{2}=C\cdot \sum_{k=1}^{K}M_{k}\sigma_{k}^{2}$$

As before, the values $\sigma_{k}^{2}$ are substituted by their definition in terms of actual measurements. Thus, we obtain for $\sigma_{wtrep}^{2}$

(15) $$\sigma_{wtrep}^{2}=C\cdot \sum_{k=1}^{K}M_{k}\left[M_{k}^{-1}\sum_{n=1}^{M_{k}}x_{k,n}^{2}-{\left(M_{k}^{-1}\sum_{n=1}^{M_{k}}x_{k,n}\right)}^{2}\right]$$

Minor transformations yield the equation

(16) $$\sigma_{wtrep}^{2}=C\cdot {\sum_{k=1}^{K}\sum_{n=1}^{M_{k}}x_{k,n}^{2}-C\cdot \sum_{k=1}^{K}M_{k}^{-1}\left(\sum_{n=1}^{M_{k}}x_{k,n}\right)}^{2}$$

Obviously, the second term in equation (16) and the first in equation (13) are identical except for the sign. Together with equations (8) and (9), this proves that $\sigma_{total}^{2}=\sigma_{wtrep}^{2}+\sigma_{\mu }^{2}$. Note that, in practice, the number of beads for each replicate is roughly equal. Hence when *M*_*k *_≈ *M *for *k *= 1,...,*K*, the weighted arithmetic averages in the above equations can be justifiably by normal averages.

## Appendix 2

### Computation of *σ*_*total *_under the condition of no systematic error

We assume the *k*-th batch-specific expression vector ${\bar{X}}_{k}$ in Table 1 to be composed of a random, batch-independent component ${\bar{Y}}_{k}$ (with standard deviation *σ*_*k *_and batch-independent mean *μ*) and batch-specific systematic shift vector ${\bar{S}}_{k}$ having equal components *s*_*k *_(thus, with mean *s*_*k *_and zero standard deviation). Whereas the standard deviation of the *k*-th replicate is not affected by the constant systematic error, the mean *μ*_*k *_is given as *μ*_*k *_= *μ *+ *s*_*k*_. Therefore, the mean *μ*^*sys *^across replicates is

(17) $$\mu^{sys}=\frac{1}{K}\sum_{i=1}^{K}(\mu +s_{k})=\mu +\frac{1}{K}\sum_{i=1}^{K}s_{k}=\mu +s$$

where *s *denotes the average of the systematic shifts. The standard deviation $\sigma_{\mu }^{sys}$ of the means from the *K *replicates is essentially the standard deviation *σ*_*s *_of the systematic shifts as is shown with the derivation

(18) $$\begin{array}{c} {\left(\sigma_{\mu }^{sys}\right)}^{2}=\frac{1}{K}\sum_{k=1}^{K}{\left(\mu_{k}-\mu^{sys}\right)}^{2}\\ =\frac{1}{K}\sum_{k=1}^{K}{\left(s_{k}-s\right)}^{2}=\sigma_{s}^{2} \end{array}$$

Consequently, the standard deviation of bead intensities $\sigma_{\mu }^{sys}$ is plagued by the systematic error. On the other hand, the standard deviation *σ*_*k *_of the *k*-th replicate is not affected by the batch-specific shift and, therefore, the batch-specific systematic error does not affect *σ*_*wtrep*_. Rightfully, the systematic error should not be present after array normalization. Hence, under the assumption of no systematic error after array normalization, usage of *σ*_*wtrep *_as reliable (lower) estimate of *σ*_*total *_is justified.

## Supplementary Material

Additional file 1 **Data for the spike experiment with Illumina BeadChips**. The tab-delimited table provides the complete summary data to re-compute the results from Table 2. Click here for file
